# Towards a better psychological satisfaction: developing a mixed multi-criteria evaluation system to urban ‘*Not in my back yard*’ facilities siting

**DOI:** 10.1186/s40359-024-01710-z

**Published:** 2024-04-15

**Authors:** Kang Cao, Xing Gao, Wei Zheng, Keyu Zhai, Jiacheng Zheng

**Affiliations:** 1 Department of Regional and Urban Planning, Zhejiang University, Hangzhou, 310058 China; 2 School of Economics, Beijing Institute of Technology, Beijing, 100081 China; 3 School of Graduate Studies, Lingnan University, Hong Kong, 999077 China; 4 Department of Urban Planning and Design, The University of Hong Kong, Hong Kong, 999077 China

**Keywords:** NIMBY facilities, Psychological resistance, Multi-criteria evaluation, Urban and regional planning

## Abstract

*‘Not in My Back Yard* (NIMBY)’ facilities are psychologically sensitive to urban and regional development. Multi-criteria evaluation (MCE) method has been widely used for the decision-making of optimum siting of urban NIMBY facilities which aim to improve residents’ psychological satisfaction. However, the evaluation of qualitative criteria in siting analysis remains under researched, such as the insufficient focus on urban and regional spatial development, social public opinion, and psychological factors. Thus, the effective improvement of MCE method through an interdisciplinary view can optimise the decision process and advance the factor assessment system of siting, which helps to supplement qualitative criteria evaluation. The specific improvement steps are as follows. The first step is to introduce the mixed MCE method to improve the qualitative criteria evaluation method by pre-processing qualitative criteria with min–max standardisation and normalization. This process transfers all negative factors to positive ones and transforms the F function to linear functions. The second step is to optimise the existing two-phase siting decision-making including the feasibility evaluation phase and the MCE phase. The third step is to propose a modular criteria system composed of urban and regional spatial planning, social psychological factors and the corresponding improvement strategy of this system from three perspectives of composition, measure, and weight. We argue that the improved method could be broadly applied to optimum siting decision of urban NIMBY facilities and enhance the psychological satisfaction of residents.

## Introduction

Recently, due to frequent mass incidents in recent years, the conflicts between urban “Not in My Back Yard” (NIMBY) facilities siting and local residents’ reactions have aroused wide social concerns. NIMBY refers to a social and psychological phenomenon that people oppose environmental facilities, infrastructure and services because of their negative externalities to be located near residents’ living places, even though they acknowledge the social necessity of those facilities [1]. Accordingly, the psychological resistance and residents’ associated behaviour is called NIMBYism or NIMBY syndrome [2]. Among the various factors evaluating NIMBY effects, the leading factors include psychological factors, such as local people’s fear of losing the perceived quality-of-life status [1], perceived environmental injustice [3], and economic factor – of losing the economic value of property [1, 4].

In general, as the facilities’ locations are main concerns of the public, there is rich literature on facilities siting of urban and regional NIMBY. For example, many social and policy studies [5–7] focus on the social conflict, policy analysis, and risk management in the process of facility siting. However, due to the limitations of siting methods, the existing literature always ignores residents’ psychological reactions. To make up the shortcomings, public and environmental psychologists try to investigate the residents’ psychological wellbeing through studying their social perception [1] and public acceptance [8, 9]. These studies suggest that siting methods of NIMBY facilities consider psychological factors. Thus, our study argues that although the siting-induced social conflict is not a pure technical issue, a scientific and reasonable siting method which takes the psychological factor into consideration can largely reduce social conflicts. To a large extent, however, the current siting and planning of NIMBY facilities are not supported by optimised technical methods, which may leave serious hidden dangers to the follow-up planning implementation. A significant manifestation is that the public often exhibits a strong attitude of criticism, dissatisfaction, and rejection towards these facilities regardless of whether they are affected by them or not. Optimum siting, namely sequencing a set of candidate sites which is usually faced by municipal governments in the urban NIMBY facilities siting and planning, is a common decision-making issue with a rational evaluation process. Multi-criteria evaluation (MCE) has been widely used in optimum siting but it heavily relies on physical criteria and overlooks social and psychological factors, especially in the early stage of NIMBY facilities. The deficient consideration on evaluation criteria variation results in the local residents’ subsequent dissatisfaction and psychological resistance, even unavoidable social conflicts.

MCE method is widely used to establish analytical models in making urban NIMBY facilities optimum siting. Previous literature can be enriched from the following three perspectives. First, they focus on the methodological multi-attribute. Keeney and Nair [10]used a multi-attribute measuring approach to study the siting of nuclear power plants. They divided the evaluation criteria into four levels and six attributes. Specifically, the health and safety level consisted of one attribute for location demographic factors; the environmental impact level contained three attributes for salmon reduction, biological impact, and cable length through environmentally sensitive areas to the 500 kV grid system; the socio-economic effect level is made up of one attribute for socio-economic impact; and the system cost level consisted of one attribute for the difference in average annual cost between locations, on which a utility function was built for analysis. Barda et al. [11] studied the siting of thermal power plants with using the Elimination et Choice Translating Reality (ELECTRE) III method in the case of EDF (Electricite de France, French Electricity) in North Africa. First, they selected 24 candidate locations according to four criteria including site area and accessibility and relevant government policy regulations such as agriculture, tourism, and infrastructure. Then, they applied the ELECTRE III method to make comprehensive decision analysis on the 20 evaluation criteria to obtain a priority order of all the candidate locations. Similarly, standing against ELECTRE III method, Norese [12] a studied the siting for waste treatment plants and incinerators in Turin, Italy.

The second perspective concentrates on insights of indicator classification, because it is a fundamentally important aspect of MCE. Erkut and Moran [13] used analytic hierarchy process (AHP) to construct a landfill siting model and studied its application to Edmonton, Alberta, Canada. They divided the factors influencing landfill siting into 3 categories: the environmental, social, and economic. Among them, environmental factors included hydrogeological, drainage, and ecological factors. Hydrogeological factors were composed of two sub-factors: surface and subsurface condition. Social factors included six factors including population, community structure, social acceptance, transportation, property value (perceived and actual effects), and other social factors. Among these factors, the community structure was composed of two sub-factors including facilities and land use conflict, and the property value factor was composed of two sub-factors, namely perceived and actual effects. Mumolo [14] also applied AHP to solve the siting of landfill by developing a corresponding analytical model in the Apulia region of Italy. Maniezzo et al. [15] explored the siting of industrial waste treatment plants using MCE method and taking the Lombardy region of northern Italy as an example. In terms of minimising cost and environmental impact, they first constructed a single objective function including transportation and construction costs, and then evaluated the prioritisation of site options based on four criteria(environmental impact, major accident risk, transportation risk, and cost), with employing the MAPPAC method. Lahdelma et al. [16] proposed a model for siting waste treatment facilities based on the stochastic multicriteria acceptability analysis with ordinal criteria and applied it to an practical project in the city of Lappeenranta, Finland. Kontos et al. [17] studied landfill site selection on the island of Lesvos, Greece, with introducing 10 criteria to exclude areas which are unsuitable for any waste disposal. Afterwards, they evaluated candidate sites with 19 criteria to select eight candidate landfills to provide a reference for government decision making. Sumathi et al. [18] discussed the construction of a landfill siting model based on the evaluation criteria such as geology, water supply, land use, sensitive sites, air quality, and groundwater quality. In the case of solid waste disposal site assessment in Arak Iran, under fuzzy environment, Ghoseiri and Lessen [19] proposed the ELECTRE approach to elaborate the impacts of human behavioural uncertainty and imprecision on site planning and developed a decision model that could better fit the real situation than other approaches.

Third, some studies stand against the geographical and spatial dimensions. Samiullah et al. [20], on considering the minimal environmental impact, proposed solid waste site evaluation criteria covering factors including distance from residential areas, groundwater level, land value, slope stability, and land use patterns. Furthermore, they combined GIS and remote sensing techniques to classify the urban area of Peshawar, Pakistan, into three types, namely the least suitable, moderately suitable, and most suitable. The landfill siting areas were thus classified as the least, moderately and most suitable. Alao et al. [21] developed a novel hybrid multi-criteria decision-making method based on Fuzzy-AHP. They further applied the method in a case of City of Cape Town, South Africa to waste-to-energy (WtE) technology selection.

However, there are two prominent shortcomings in the current MCE methodology. First, qualitive criteria are under-considered. Because it is difficult to quantify qualitative criteria, like public perception and psychological health risks and etc., previous studies prefer to develop an mathematical model based on the quantitative criteria. Thus, they tend to employ quantitative criteria evaluation methods and ELECTRE method. However, insufficient considerations of qualitative criteria lead to the mismatching between the analytical model and the reality. In particular, political and social factors are solely and cursorily considered in the final approval stage, resulting in the local residents’ psychological dissatisfaction and opposition to many siting decisions [22]. In addition, the lack of qualitative standards results in significant resistance for experts with engineering and technical backgrounds when setting the composition and weight of evaluation standard types [23]. Thus, the phenomenon requires specific attention when applying the MCE method. Second, partial optimisation deficiencies exist. Researchers applying the MCE method for urban NIMBY facilities siting are mostly from the engineering, environmental science, and operations fields. Correspondingly, their studies are mainly based on the technical and economic data of the facilities. However, they rarely consider the complexity of urban spatial interactions between facilities and urban spaces, the dynamics and uncertainty of urban spatial and temporal development. Furthermore, they usually overlooked the negative psychological consequences of inappropriate sitting of NIMBY facilities on the public. Concisely, when applying the method of MCE method, researchers always must address the problem of combining quantitative evaluation and qualitative criteria. Thus, the evaluation method which is improved by combing qualitative criteria and developing comprehensive considerations will lead to a optimum siting.

Accordingly, we aim to develop a mixed MCE method to respond to the issues and to improve psychological satisfaction of the public to the NIMBY facilities siting. The study contributes to the existing knowledge in the following ways. First, we use the algorithm optimisation of F function to deal with the evaluation issue of qualitative criteria. Specifically, the study transfers it from a signum function into a linear function. By doing so, our method takes full consideration of and quantifies the qualitative criteria, an aspect which existed methods lack consideration, to overcome the first shortcoming of exist studies. In detail, we preprocess the qualitative indicators by using min max standardization and normalization, and perform forward normalization on all negative indicators. The calculation results of F function also exhibit different numerical values, which can effectively distinguish the advantages and disadvantages of different combinations of qualitative criteria. Overall, this study optimizes the quantitative evaluation problem of qualitative criteria, thereby improving the scientificalness of the psychological satisfaction evaluation model.

Second, we optimise the decision process and establish a modular criteria evaluation system. Aiming at psychological satisfaction improvement, our evaluation criteria system takes the four modules, namely the spatial planning, environment protection, construction-operations, and socio-psychology module into equally consideration. It thus resolves current studies’ shortcoming of overlook the complicated urban spatial interaction and dynamic, and the negative psychological consequences. The mixed MCE developed can potentially improve the scientific and operability of multi-site merit decision for urban NIMBY facilities and increase the possibility of public acceptance. In addition, we will combine the optimized decision-making process with these four modules. Especially, this study designs a comprehensive site selection evaluation criteria system by focusing on the composition, measurement, and weight of the criteria. Therefore, by focusing on the actual situation of multi-site optimization decision-making, the psychological satisfaction of the affected is improved.

Three sections follow the Introduction section. Psychological satisfaction of NIMBY section shows a literature on psychological satisfaction of NIMBY. In Materials and methods section, we develop our method, including optimising the mixed multi-criteria method and improving the siting evaluation system. Empirical analyses of MMCE section will apply the methodology developed for a case study. Finally, we conclude our study in Conclusions section.

## Psychological satisfaction of NIMBY

Residents' psychological satisfaction of NIMBY facilities is constituted by multiple dimensions of perception, including risk perception, benefit perception, justice perception, etc. Risk perception is individuals' attitudes and intuitive judgments towards risks. It is the perception formed during the evaluation process of the characteristics, uncertainties, and harms of objectively existing risk facts in uncertain situations, under the subjective influence of various factors in their social environment. Similarly, it is a core concept for understanding the public's response to NIMBY risks and a fundamental factor in shaping the public acceptability of NIMBY facilities. Numerous studies have already found a negative correlation between residents' risk perception of NIMBY facilities and the public acceptability of these facilities. There are two types of risk perceptions associated with NIMBY facilities. One of them is cognitive components of perceived risks, which involves the definition and conceptualization of the risk perception concept, based on the cognitive aspects of NIMBY facility risk perception. For example, Liang et al. [24] found a positive correlation between facility safety perception and public acceptance of nuclear waste treatment facilities. Additional, Lober [25] found that risk perception significantly predicts public acceptability of four types of waste avoidance facilities, including landfill sites, recycling stations, waste transfer stations, and waste incineration facilities, when analyzing public attitudes towards the. The second type is affective risk perception, which involves the affect theuristic as heuristics in the formation of risk perception [26].

Benefit perception refers to the perception of material benefits or losses by residents near NIMBY facilities. Loss of benefits includes depreciation of property values, deterioration of investment environment, and potential harm to physical health [27]. While benefits are the convenience or welfare provided to the public in meeting their living needs by the NIMBY facility itself as a public good. This includes the creation of new employment opportunities locally, the generation of local fiscal revenue, and even significant contributions to the promotion of local economic development [28]. Many studies have found that benefit perception is an important predictor of public acceptability of NIMBY facilities [9, 29]. A significant number of domestic and international scholars have analyzed the impact of perceived justice on the public acceptability of NIMBY facilities, focusing on distributive justice and procedural justice [30]. Similarly, Batel and Devine‐Wright [31] found that a lack of justice is a significant reason for community residents opposing wind power plants. In addition, some studies have divergent opinions on how to address the issue of justice deficiency in the negotiation process. For example, Besley [32] found that procedural justice and distributive justice positively and significantly influence the acceptance of expansion project decisions by surrounding residents, with no significant differences in the impact effects. The results also indicate that anger emotion negatively moderates the relationship between procedural justice, distributive justice, and residents' acceptance [32]. Some scholars have found that the impact effects of distributive justice and procedural justice on the acceptability of NIMBY facilities differ. For example, Walter [33] found that procedural justice and distributive justice simultaneously significantly influence residents' acceptance of wind power plants. However, the impact of procedural justice is relatively small, while distributive justice has a very noticeable effect on public acceptability [33]. Lima's research results indicate that distributive justice positively and significantly influences public acceptability, while procedural justice has no significant impact on public acceptability of an incineration facility. Further analysis results show that distributive justice partially mediates the relationship between distance and public acceptability of the incineration facility [34].

In addition, some indirect costs of mental disorders problems can be led by NIMBY facilities. Concerns about environmental impact, pollution, and potential health risks, which are always link with the NIMBY facilities, can create a stressful environment in the community, contributing to heightened levels of and chronic anxiety among the local residents. Chronic anxiety or stress is widely known as an apparent risk factor for many mental disorders. In addition, NIMBY facilities are economic stressors as they may cause the decline of the surrounding communities' property values and lead to economic insecurity and significant anxiety for homeowners who see their home as a major investment or a source of financial security. The mental disorder problems generated by these factors will no doubt lead to indirect costs. Commonly, the indirect costs include but not limited to: 1) increasing healthcare costs by more frequently using healthcare services or even hospitalizations; 2) more demand for social or support services like social workers and community care; 3) reduced opportunities for property investment of the affected communities and regions; and, 4) decreasing quality of life, productivity and social participation of the affected local, which may cause substantial economic impact on the society and economy.

## Materials and methods

### Algorithm improvement for Mixed MCE

**Fig. 1:**
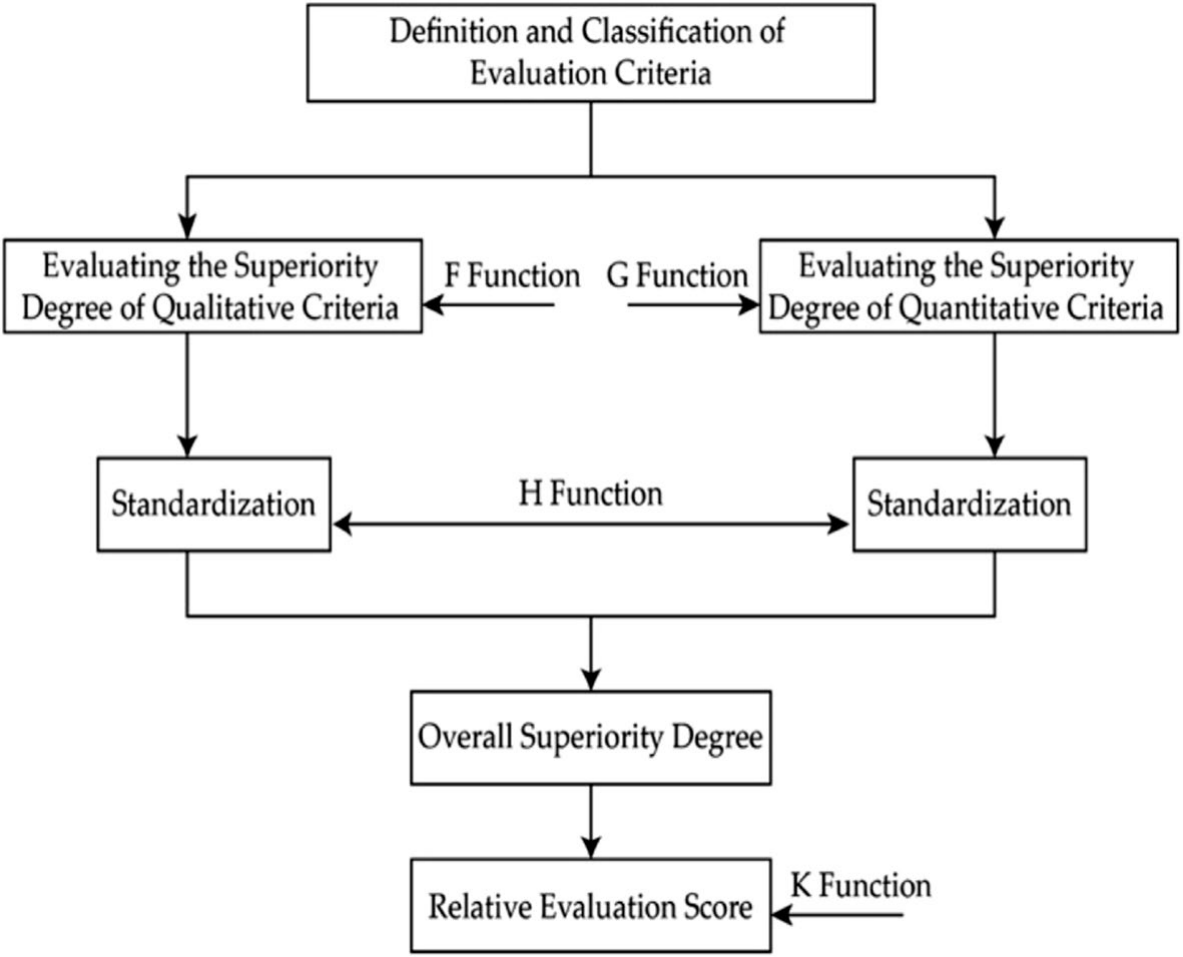
Analysis process of MEQQD

We develop the mixed MCE on the ground of multi-criteria evaluation mutually supported by qualitative and quantitative data (MEQQD), which was proposed by Dutch scholar Voogd in 1983 [35]. The evaluation process effectively and comprehensively takes both qualitative and quantitative criteria into account (Fig. 1). Traditional method is nevertheless not applicable for NIMBY facilities siting in urban and regional contexts because of its conspicuous defects of F function. In detail, when evaluating the superiority degree of qualitative criteria, F function fails to assign values to qualitative criteria, as analysed below.

The F function used in MEQQD to measure the superiority of qualitative criteria is:

1 $${a}_{ij}=\overline{F}\left({e}_{ik},{e}_{jk},{w}_{k}\right)={\left\{\sum {\left[{w}_{K}\left({e}_{ik}-{e}_{jk}\right)\right]}^{r}\right\}}^\frac{1}{r},k\in O$$

Where ${a}_{ij}$ stands for the extent how $i$ scheme is superior to $j$ scheme; $r$ is the parameter that adjusts the weight reliability; ${w}_{k}$ indicates the weight of the criterion $k$. The $\mathit{sgn}\left(x\right)$ function means:

2 $$\mathit{sgn}\left(e_{ik}-e_{jk}\right)=\left\{\begin{array}{ccc}1&,&if\;e_{ik}\rangle e_{jk}\\0&,&if\;e_{ik}=e_{jk}\\-1&,&if\;e_{ik}\langle e_{jk}\end{array}\right.$$

**Fig. 2:**
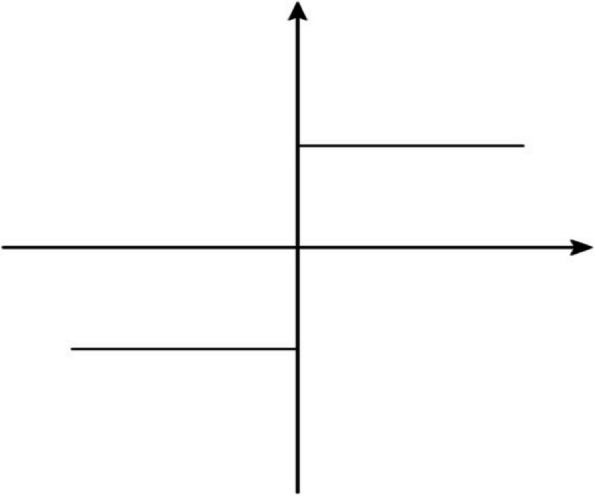
The graph of signum function

The graph of signum function $\mathit{sgn}\left(x\right)$ is shown in Fig. 2. The ${a}_{ij}$ value is influenced by the criterion weight ${w}_{k}$. Their positive or negative signs are from the signum function $\mathit{sgn}\left(x\right)$, but they are uncorrelated to the actual value of $\left({e}_{ik}-{e}_{jk}\right)$. Consequently, it cannot distinguish the superiority of different options for NIMBY facilities siting and must be improved by enabling the value of $\left({e}_{ik}-{e}_{jk}\right)$ to influence ${a}_{ij}$. In this way, the F function can evaluate the superiority of the combinations of qualitative criteria.

**Fig. 3:**
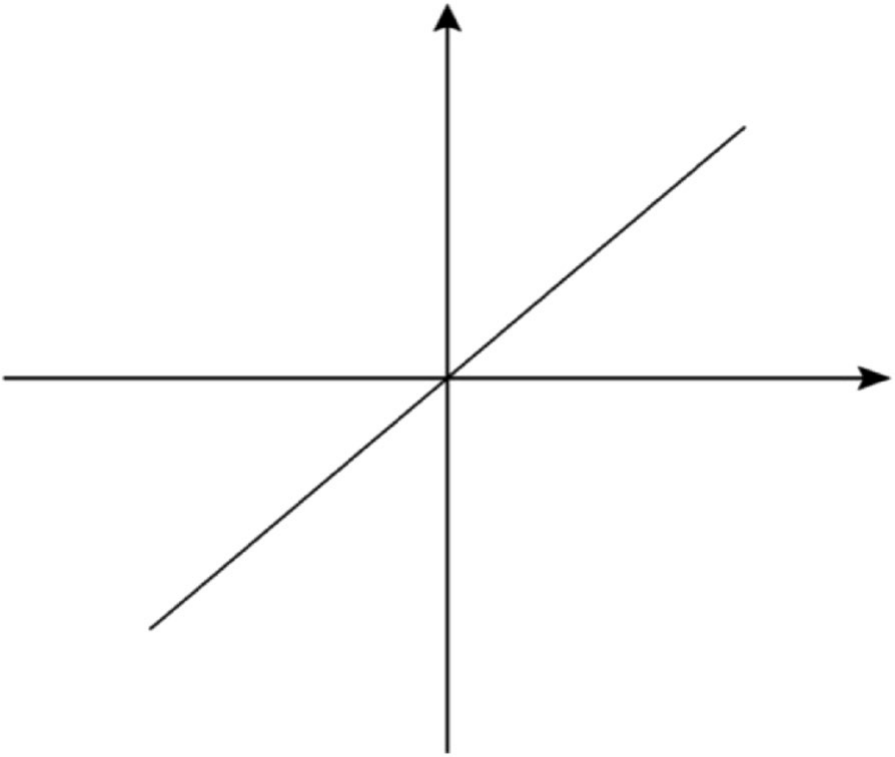
The graph of linear function

The improved MCE method introduces the standardization and normalisation processing (Eq. 3), transfers all negative indexes into positive ones (Eq. 4), and replaces the signum function $\mathit{sgn}\left(x\right)$ with linear functions (Fig. 3). The formulas are as follows (Eq. 5):

3 $$\overline{{e}_{ji}}=\frac{{e}_{ji}-\mathit{min}({e}_{j})}{\mathit{max}({e}_{j})-\mathit{min}({e}_{j})}$$

4 $${{e}^{\mathrm{^{\prime}}}}_{ik}=\left\{\begin{array}{c}{{e}^{\mathrm{^{\prime}}}}_{ik}, if\,positive\\ 1-{{e}^{\mathrm{^{\prime}}}}_{ik}, if\,negaive\end{array}\right\}$$

5 $${a}_{ij}=\overline{F}\left({{e}{\prime}}_{ik},{{e}{\prime}}_{jk},{w}_{k}\right)={\left\{\sum {\left[{w}_{K}\left({{e}{\prime}}_{ik}-{{e}{\prime}}_{jk}\right)\right]}^{r}\right\}}^\frac{1}{r},k\in O$$

where r is the scaling parameter and specified as an odd number. The lower the reliability of the weights is, the larger the value of $r$ should be. The calculation of $\overline{F}$ accordingly has different results as the value of $\left({{e}^{\mathrm{^{\prime}}}}_{ik}-{{e}^{\mathrm{^{\prime}}}}_{jk}\right)$ varies, proving that the improved mixed MCE can distinguish the superiority and inferiority of different combinations of qualitative criteria.

### Siting decision-making assessment process optimisation

**Fig. 4:**
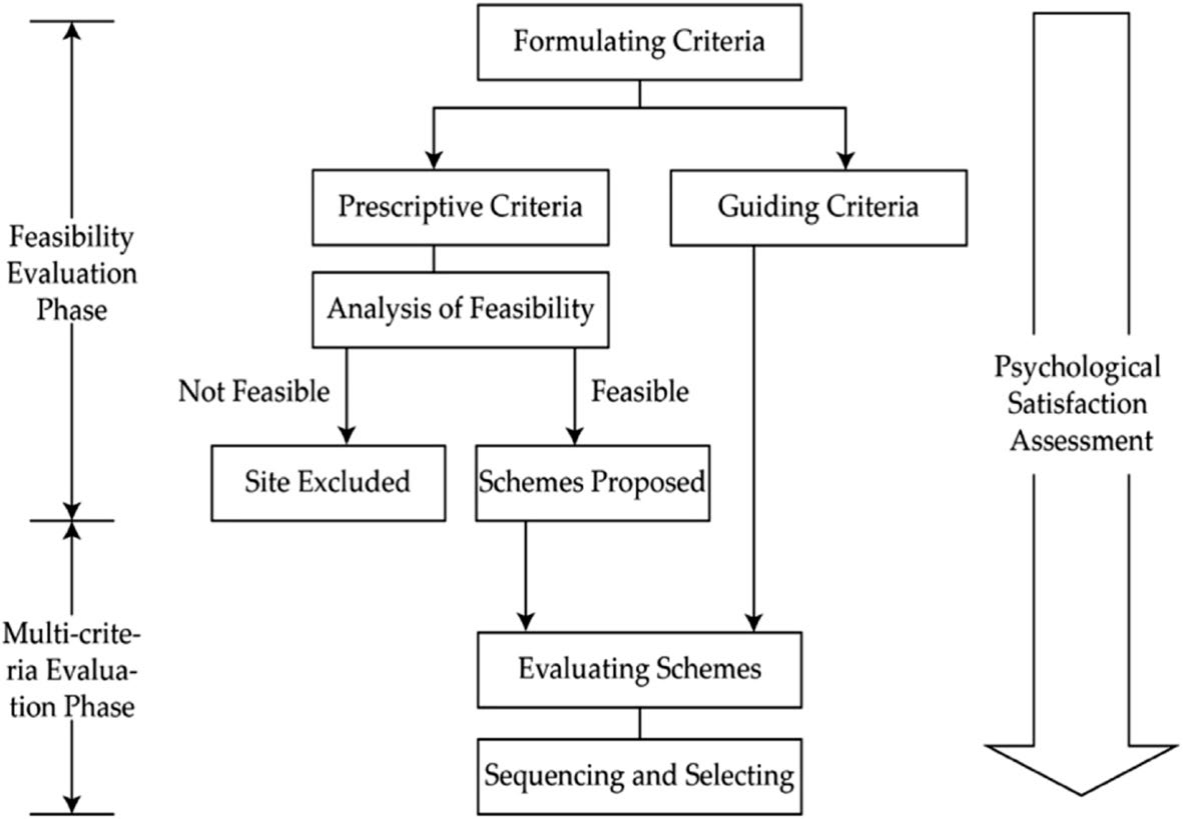
Decision and psychological satisfaction assessment process of MCE in NIMBY facilities siting

Applying MCE to urban NIMBY facilities siting is a process of selection and prioritisation. The process is also regarded as a decision-making phase where all criteria are placed at the same hierarchies for evaluation and analysis [13, 36]. However, we argue that adding a feasibility evaluation phase before conducting the MCE will largely enhance the reasonability of the decision-making and increase the possibility of public acceptance. Accordingly, the process can promote psychological satisfaction. Thus, the process will be consisted of two phases including the feasibility evaluation phase and the MCE phase. The feasibility evaluation is a preliminary screening phase to confirm a potential site through excluding prohibited ones, such as sites that conflict with the ecological protection requirements, and sites with geological conditions that do not meet construction requirements. We can classify the evaluation criteria into two types including prescriptive and instructive. The prescriptive criteria are applied to evaluate the feasibility phase, indicating a ‘yes’ or ‘no’ relationship. Only sites that meet the requirements of the prescriptive criteria can proceed to the next phase of MCE (Fig. 4). The instructive criteria are applied to the MCE phase and are involved in the quantitative analysis. The two-phase evaluation process design not only conforms to a logical thinking of planning profession, but also better fits the reality of optimum siting decision-making than the one-phase process. Moreover, it optimises the psychological satisfaction assessment process.

### Siting evaluation criteria system improvement

**Fig. 5:**
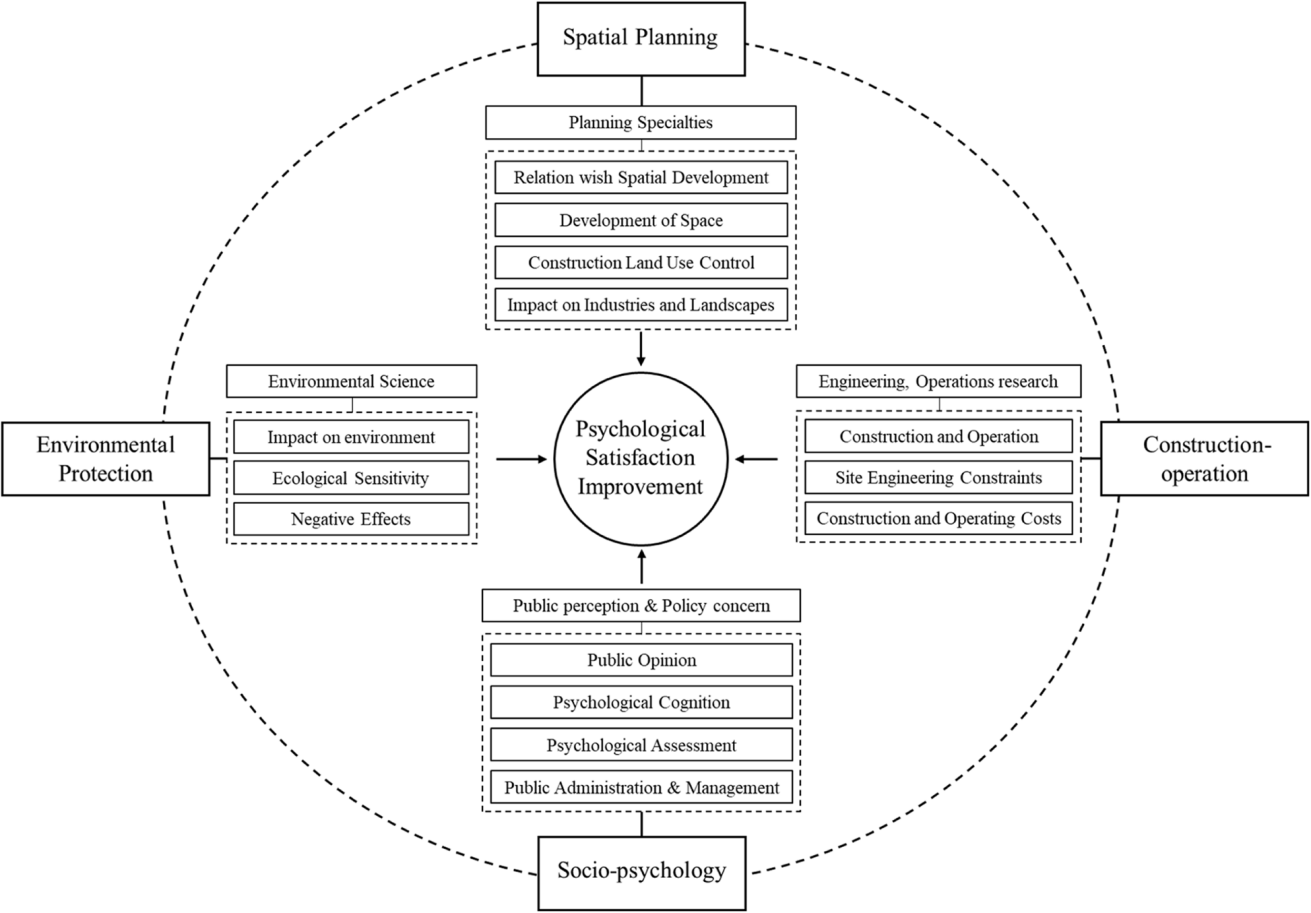
Four modules of MCE system to improve psychological satisfaction

We attempt to improve the siting evaluation criteria system and thereby promote the public psychological satisfaction which is affected by the composition, measure, and weight of NIMBY facilities. Firstly, in terms of composition, the deficiencies of the composition criteria are mostly caused by the insufficient cross-domain collaboration and the excessive focus on engineering content. Such causes together with the difficulty in quantification lead to insufficient consideration of socio-psychological dimensions, such as the public perception and the impact of facilities on spatial development [23]. Considering the complexity of siting criteria, we develop a criteria system through modularisation. Modularisation means that corresponding research fields or disciplines and evaluation contents are closely related to generate various modules which strengthen interdisciplinary collaborative decision-making. Such modularisation process can shrink the bias caused by single-disciplinary decision-making. According to the analysis of the planning and construction conditions of NIMBY facilities, the evaluation criteria systemcan be developed through four modules, i.e., spatial planning, environmental protection, construction-operation, and socio-psychology (Fig. 5). The spatial planning module mainly falls within the scope of urban and regional planning specialties. It is configured from the relationship between the NIMBY facilities and urban and regional spatial development. In detail, we should notice the possibilities of facilities’ compatibility to the development direction of urban and regional space and adaption to requirements of construction land use control and the impact of the facilities on the surrounding industries and landscapes. Environmental protection module mainly falls into the category of environmental science and focuses on the specific impact of NIMBY facilities on the surrounding environment. It should be noted whether the facilities meet spatial control requirements of ecological sensitivity and whether there exist negative effects such as air pollution, water pollution, electromagnetic radiation, nuclear radiation, noise, explosion risk and etc. Construction-operation module covers engineering, operations research, and other related disciplines and evaluates the construction and operation of adjacent facilities from the technical and economic perspective. Site engineering constraints, construction costs, and operating costs are all included. Socio-psychology module involves public opinion, psychological cognition, psychological assessment, public administration and management, and mainly considers public perception and policy concern.

The following issues should be given timely and extensive attention in formulating specific criteria. First, in the spatial planning module, we cannot ignore the impact of NIMBY facilities on the surrounding industries and landscapes. Especially, high-tech industrial clusters, cultural sites, and scenic spots around the facilities cannot be ignored because NIMBY facilities are psychologically sensitive to urban and regional development. Second, in the environmental protection module, although there are no clear scientific research findings, or safety standards, environmental risks should be incorporated into the evaluation criteria system. Third, the construction cost of peripheral supporting infrastructure should be deemed in the construction-operation module. Forth, in the socio-psychology module, it is essential to consider the feasibility of setting up socio-psychology criteria regarding residents’ perception, reaction, and satisfaction at public policy level. When it comes to the significant urban NIMBY facility siting, particularly, decision-makers can get involved into the model analysis earlier if the policy concerns are incorporated. It also helps to reduce the ineffective repetition in decision-making process. Fifth, a precise distinction needs to be made between prescriptive and instructive criteria for each module. This distinction is particularly important for the socio-psychology module where experts tend to regard siting as a soft constraint from the socio-psychological aspect. They ignore the decisive impacts of diverse cultural situations, moral values, and psychological cognition, and thus there is few adequate attention and vigilance in setting prescriptive criteria for this module. As Walter Joos, et al. [37] have strengthened these aspects including the social, psychological, and political issues such as public participation in planning and implementation, consumer behaviour, and changing value systems, which are as important as the technical and economic aspects of neighbourhood planning decisions.

Secondly, regarding measure of criteria, it is applied to deal with the quantification issue of instructive criteria because they are indicators to assess the ranking of each feasible option in the model calculations. There are several issues as follows. The first is the measure of quantifying criteria. Among the four modules, the instructive criteria for environmental protection module and construction-operation module are quantitative, and they have relatively mature approaches to measure. However, it should be noted that the siting decision-making process often does not involve construction methods and techniques. The negative environmental impact can only be judged in terms of technical regulations and engineering context, and it cannot be supported by the accurate data. Regardless of the technical level or the decision-making cost and efficiency level, excessive pursuit on measurement accuracy is not applicable to such case. Adopting alternative methods is more applicable, such as shortening the distance between facilities and infrastructure rather than reducing the direct construction cost of the facilities,. For instance, the construction cost of supporting infrastructure can be reduced when shortening the distance between NIMBY facilities and nearby water supply and drainage and power systems However, we should also pay attention to the indirect costs caused by mental disorders problems which are generated from the NIMBY syndrome.

The second is the measure of qualitative criteria. Qualitative criteria primarily centre on spatial planning and socio-psychology modules. Common qualitative criteria include impacts of public, industrial and landscape and assessment on public perception and psychological satisfaction. It should be removed of the qualitative criteria which lack accuracy. Although the impact of NIMBY facilities on surrounding industries can be evaluated quantitatively, qualitative evaluation is a more convenient and desirable way. The convenience stems from the dynamics and uncertainty of urban and regional spatial development, residents’ psychological perception, as well as the difficulty and high cost of quantitative evaluation itself. The practical operation of qualitative criteria can be conducted by conventional attitude scale method. We argue that researchers and practitioners should fully consider the urban and regional space dynamics when marking values for qualitative criteria. The logic is that the NIMBY facilities siting is closely contingent on the urban and regional spatial development, as well as its overall planning. Thereby, the researchers and practitioners need to comprehensively analyse and estimate the trend of land use and population distribution.

They should also avoid the undesirable tendency of simplistic quantification. Such quantification is exemplified by using the direct ‘headcount’ approach to conducting public psychological perception evaluation criteria. Two causes can show why such approach should be avoided. First, social, psychological, political, economic, and cultural factors play important roles in risk perception and directly affect public attitudes and psychological satisfaction [38]. Second, large differences exist among communities with respect to political-economic power and organisation capacity, which influences the degree of public acceptance, strength of public psychological reaction and public policies’ effectiveness. Findings on public participation in NIMBY facility planning further reveal divergent attitudes incurred by the population composition differences in communities. In the case study of wind energy projects in Sweden, Devlin [39] found that the proportion of permanent or temporary residents in all the residents affects communal acceptability of wind energy projects. If temporary residents dominated the community, residents’ psychological dissatisfaction would be increased and community resistance to proposals would be strengthened.

The third issue is about weight study. Weights reflect the decision-makers’ judgment on the importance of the instructive criteria and also are substantive factors when ranking the schemes. Although the specific measure of weights might vary on NIMBY facilities’ types and spatial characteristics, some common issues need to be taken into consideration. In terms of cross-domain collaboration, under the prolonged influence of technological rationality, experts with engineering and technical background tend to have a greater voice and influence on weight setting than those with humanities and social sciences background. This situation results in the lack of adequate attention to the public psychological perception and assessment in the process of siting decision-making [23]. However, urban NIMBY facilities siting is more of a social and psychological issue which influences residents’ psychological perception and reaction than a technical one. Therefore, criteria weighting in the spatial planning and socio-psychological modules should be conducted through cross-domain collaboration. Regarding public participation, due to the technical thresholds and sensitivity to interest, the siting decision-makers are often reluctant to involve the public in the decision-making process. Thus, they ignore the public psychological perception and assessment. Experts who apply the traditional decision-making models with technological rationality view can only observe the socio-economic and cultural situation in a superficial way and believe that they have made scientific analyses and judgments. However, the process could trigger problems of correlation and feasibility [40] and reduce public satisfaction. As Coleby et al. [41] have pointed out, wind energy projects were often questioned by physical factors such as noise, light pollution, and killing birds. However, community residents’ psychological perception and assessment, which were revealed through public participation process, often focused more on psychological issues, such as the visual impact of the NIMBY facilities [17]. Therefore, effective public participation can rectify the deviation of decision making, make sure that the weights are closer to the real public psychological perception, and improve the reasonability of the siting evaluation model building. Finally, public satisfaction can be promoted.

Regarding weight determination methods, they include psychological, objective, and comprehensive weight-assignments. Currently, psychological and objective weight-assignments are generally applied. On the one hand, while psychological weight-assignment can better reflect the intention of decision-makers, it has greater arbitrariness and uncertainty than the objective one. On the other hand, despite the objective weight-assignment has a stronger mathematical and theoretical basis than the psychological one, it sometimes contradicts the actual importance ranking of each criterion and fails to reflect the psychological assessment and judgment of decision makers. In view of the complexity of NIMBY facilities siting evaluation, comprehensive weight-assignment which integrates the psychological and objective ones is a better option to improve the rationality of the weight assignment. This process considers both the empirical judgment of policy makers and the inherent rules between the various criteria. The formula of mixed weight-assignments is as follows (Eq. 6):

6 $${w}_{i}=\alpha {a}_{i}+\left(1-\alpha \right){b}_{i}, (0\le \alpha \le 1)$$

where ${w}_{i}$ represents the portfolio weight of $i$-criteria; ${a}_{i}$ and ${b}_{i}$ respectively represent the objective and psychological weight of $i$- attribute. When decision makers have preference for different weight-assignment methods, α can be determined according to the decision maker's preference.

Objective weighting evaluation utilises variation coefficient method. The basic principle of this method is to take the indicators with the greater variation, i.e., indicators which are difficult to be concluded and synthesized. The indicators can reflect the gaps between the evaluated objects. It is necessary to pre-process the indicators through the variation coefficient formula (Eq. 7) to eliminate the influence of differences between various indicators’ dimension:

7 $${V}_{i}=\frac{{\sigma }_{i}}{\overline{{x }_{i}}} (i=\mathrm{1,2},\cdots ,n)$$

where ${V}_{i}$ is the variation coefficient of item $i$ indicator, also known as the standard deviation coefficient; ${\sigma }_{i}$ is the standard deviation of item $i$ indicator; $\overline{{x }_{i}}$ is the average of item $i$ indicators.

The weights of each indicator are calculated as follows (Eq. 8):

8 $${a}_{i}=\frac{{V}_{i}}{\sum {V}_{i}}$$

The psychological-weight assignment method uses Delphi method. Specifically, the weight ${b}_{i}$ is obtained through the relative importance assigned by the selected experts to each evaluation indicator.

## Empirical analyses of MMCE

### Case study

We chose Hangzhou as the case study, which is a city located in the southeast coastal areas of China. In recent years, a rapid pace of urbanisation had seen the increase of household waste production at the rate of about 10% per year. The situation could hardly be improved because of the current way of sanitary landfill. To reduce waste and encourage recycle resources, the municipal government planned to employ waste incineration to deal with the problem of household waste exponentially increases. According to the relevant planning and land conditions, the related municipal department initially selected five candidate sites for a waste incineration plant and made prioritising analysis to improve the residents’ satisfaction and to avoid conflicts.

In terms of the planning and land use condition, the government department had commissioned a research institute to make siting analysis. The research institute adopted the AHP to develop a three-layer (goal, criterion, and index layers) incineration siting criteria evaluation system. The layer of goal was the incineration site’s applicability. The layer of criterion consisted of four influencing factors including engineering geographical condition, environment protection condition, incineration plant construction condition, and transportation condition. Each factor incorporated a set of evaluation index which integrated the index layer. For example, the plant construction condition incorporated slope gradient, storage capacity, land cost, the distance away from the existing roads, and water and power supply facilities.

After analysis, the institute concluded five candidate sites’ priority orders. However, the government authority was dissatisfied with the research conclusion in that the conclusion was drawn from case-by-case research because the research did not consider the urban spatial development layout, historical culture and landscape protection, and the acceptance and satisfaction of the public complicated qualitative criteria, such as. Thus, these conclusions have major defects. Given all this, the authority commissioned us to restudy the waste incineration siting issue. We then adopted this siting analysis method and made the siting analysis through two phases of feasibility evaluation and MCE.

### Results

Firstly, in the stage of feasibility estimation, we developed a waste incineration plant siting evaluation criteria system (Table 1) for each of the afore-mentioned four modules through two steps. In the first step, we initially selected the evaluation criteria along with a comprehensive theoretical analysis and a Delphi method. In the second step, we further employed a principal component analysis method to screen the selected evaluation criteria with. In this case, we could determine the prescriptive criteria through the other three modules as there were no socio-psychological constraints.

**Table 1:** The development of the siting evaluation criteria system for waste incineration plants

Modules	Types	Evaluation criteria	Description	Standard metrics	Nature
Spatial planning	Prescriptive criteria	Land use	Whether use and scale of the land use meets the requirements of land use control and design	—	—
		Relationship with spatial development	Whether the siting is in line with the future development goals of urban and rural space, whether there is a security risk	—	—
	Instructive criteria	Public impact^a^	The degree to which the facilities affect the physical and psychological health of the surrounding residents and the value of real estate	Evaluated by a Likert Scale with 1–5 points	Qualitative criterion
		Land size	Whether the site has room for future development	Site land area (units: square meters)	Quantitative criterion
		Industrial impact	The adverse effects of facilities on surrounding industries	Evaluated by a Likert Scale with 1–5 points	Qualitative criterion
		Landscape impact	The adverse effects of the facility on the surrounding landscape	Evaluated by a Likert Scale with 1–5 points	Qualitative criterion
Environmental protection	Prescriptive criteria	Ecological environment sensitivity	Whether consistent with the regulatory requirements for natural, ecological and environmentally sensitive areas	—	—
	Instructive criteria	Atmospheric environmental impacts	Evaluation the extent of the impact on local air pollution levels	Value added of atmospheric environmental pollution (in ppm)	Quantitative criterion
		Noises	Assess the extent of the impact on local noise	Value added (in decibels) based on background noise level	Quantification criteria
Construction-operations	Prescriptive criteria	Terrain	Whether the site meets terrain-defining conditions	—	—
		Geology	Whether the site meets the conditions stipulated in engineering geology	—	—
	Instructive criteria	Land costs	Land costs required for construction, including sites and supporting land (e.g. road acquisition)	Land collection fees, including demolition compensation costs (units: 10,000 CNY)	Quantitative criterion
		Construction investment	Incinerator construction costs	Includes site levelling, facilities and engineering construction costs (units: 10,000 CNY)	Quantitative criterion
		Infrastructure support	The costs associated with the supporting of infrastructure such as water, electricity and roads	Distance from site to water, electricity and road infrastructure (in meters)	Quantitative criterion
		Shipping costs^b^	The transportation cost of domestic waste to the incinerator for disposal, and of residues to landfills after incineration	Distance between incinerator and landfill (in meters)	Quantitative criterion
		Running costs	The cost of management, operation, etc	Including waste incineration treatment, equipment maintenance, daily management costs (single: 10,000 CNY)	Quantitative criterion
Socio- psychology	Instructive criteria	Public perception	Public opposition around the facility is under pressure	Evaluated by a Likert Scale with 1–5 points	Qualitative criterion

In the public impact, industrial impact, and landscape impact assessments, the degree of impact is proportional to the size of the score. For the public perception assessment, the pressure from public opposition is proportional to the size of the score

^a^Criteria for public impact. If the site’s spatial features are relatively homogeneous, the criterion can also be measured by population and residential land area. Accordingly, the nature of the criterion is transformed into a quantitative criterion. We applied qualitative evaluation in this case because the spatial features were largely heterogeneous and considered the surrounding area’s future development. This also indicates that different measures need to be taken appropriate to the real situation in the specific criteria assessment

^b^Transport costs criteria. The transportation cost of incineration plant mainly includes two aspects. One aspect is the transportation cost of garbage collection and transportation to incineration plant and the other is the transportation cost of transportation of residue after incineration treatment to landfill for sanitary landfill. This cost is measured by the distance of transport, namely the distance converted into garbage collection and transportation and the distance of the debris cleared to landfill. Due to objective conditions, the transportation cost in this case study is measured only by the distance from the landfill to where the residue is collected

In terms of determining to apply the Delphi method, we solicited experts’ opinions rather than a broad range of the residents’ opinions. If we could solicit residents’ opinions in the prophase siting study period, the rationality and scientificity of Delphi method could be improved, and also its analysis on the psychological factor of the residents could be strengthened. In the real case, however, information leakage in the prophase study period would trigger psychological resistance and the associated resident behaviours, like mass resistance incident. Therefore, relevant government authorities required that the research team did not broadly solicit the residents’ opinions during this period. However, we did collect sample residents’ opinions with the Delphi method. The expert opinions were solicited by mails. We sent out 60 mails and received 57 valid ones back. The experts’ research domains covered environmental engineering, urban planning, public management, sociology, ecology, and psychology. Their affiliations included local government and affiliated municipal departments, planning and design consultant agencies, universities, NGOs, and communities’ residents where the candidate sites were located. Experts had a strong consensus on the need to increase the qualitative criteria. They also generally agreed on each module’s specific criteria setting. Their key dispute was the weight issue. Experts from environmental engineering industry, relevant municipal departments, and design agencies assigned significantly higher weights to environmental protection and construction-operation modules than experts from other research domains and affiliations, who laid more emphasis on the spatial planning and socio-psychological modules.

The second is candidate’s evaluation and merit analysis. Based on the prescriptive criteria selection in the three modules of spatial planning, environmental protection, and construction-operation, three site schemes were finally identified for the MCE phase. The criteria for the three candidate site schemes were measured as follows (Table 2).

**Table 2:** Comparison of the basic conditions of the three waste incineration plants siting schemes

Modules	Evaluation criteria	Scheme 1	Scheme 2	Scheme 3
Spatial planning	Public impact	4	3	2
	Land size (sqm)	53300	57600	60400
	Industrial impact	3	3	2
	Landscape impact	1	2	1
Environmental protection	Atmospheric Environmental Impact (ppm)	0.4	0.6	1
	Noise (decibels)	8	8	5
Construction-operations	Land cost (10,000 CNY)	1020	920	870
	Construction investment (10,000 CNY)	33500	35300	35500
	Infrastructure investment (m)	1540	2520	3680
	Transportation cost (m)	9800	11500	14400
	Operation cost (10,000 CNY per year)	1560	1650	1680
Sociopsychology	Public perception	4	4	2

Qualitative criterion data are determined by the Delphi method and quantitative criterion data are provided by relevant departments

Then, we conducted data preprocessing. Evaluation criteria were divided into two categories, namely qualitative criteria and quantitative criteria, which could be represented by ‘O’ and ‘C’, where:

O = {public impact, industrial impact, landscape impact, public perception}, and is the set of all qualitative criteria

C = {Scale of site, atmospheric impact, noise, land cost, construction investment, infrastructure support, transportation cost, operation cost}, and is the set of all quantitative criteria

The min–max method was used to normalise and standardise quantitative and qualitative data and to eliminate the impact of quantitative framework. Thus, negative indicators were normalised to convert into positive indicators. Specifically, the site size in the siting criteria was a positive indicator, while the rest were negative indicators. The weighting values for each evaluation criterion were obtained according to the integrated psychological and objective weighting method. The results were calculated as follows (Table 3).

**Table 3:** A list of evaluation criteria for weight assignments

Evaluation Criteria		Expert’s weight-assignment	The weight of the coefficient of variation	The combined weight ɑ = 0.5
Quality criteria	Public impact	0.18	0.0784	0.1292
	Industrial impact	0.1	0.0679	0.0839
	Landscape impact	0.02	0.0000	0.0100
	Public perception	0.15	0.1357	0.1429
Quantification criteria	Land size	0.08	0.0738	0.0769
	Atmospheric environmental impacts	0.12	0.0718	0.0959
	Noises	0.08	0.1357	0.1079
	Land costs	0.05	0.0718	0.0609
	Construction investment	0.03	0.1177	0.0739
	Infrastructure support	0.05	0.0763	0.0632
	Shipping costs	0.08	0.0729	0.0765
	Running costs	0.06	0.0979	0.0789

Third, we implement mixed MCE. We made superiority analysis of the evaluation criteria, normalised the degree of superiority and calculated the overall superiority level to obtain (Eq. 9):

9 $${m}_{ij}={W}_{o}{b}_{ij}+{W}_{c}{d}_{ij}$$

where *m*_*ij*_ is the overall degree of superiority of Scheme 1 (S_1_) to Scheme 2 (S_2_); *W*_*o*_ is the sum of all the qualitative criteria’s weights; *W*_*c*_ is the sum of all the quantitative criteria’s weights; *b*_*ij*_ is the normalizing result of qualitative degree of superiority; *d*_*ij*_ is normalizing result of the quantitative degree of superiority. Thus, we obtained (Table 4):

**Table 4:** The calculation result of the schemes’ overall degree of superiority

	**S1**	**S2**	**S3**
**S1**	0	0.215196799	0.25
**S2**	0.215196799	0	0.034803201
**S3**	-0.25	0.034803201	0

We then compared the three schemes (Eq. 10).

10 $${S}_{i}={M}_{i}=\sum_{j}{m}_{ij}$$

And thus we obtained: S_1_ = 0.4652, S_2_ = -0.1804, S_3_ = -0.2848. The evaluation result was that Scheme 1 was better than Scheme 2 and Scheme 3. This result was then adopted as an important basis for the local government’s siting decision-making to improve the psychological satisfaction of the affected.

### Discussion

There exist major differences, which are derived from this article’s case study of site selection analysis comparison, between the AHP and the mixed MCE method. First, AHP made priority order for all the five candidate site schemes, whereas the mixed MCE ruled out two candidates which obviously contradicted with the urban special development priority and the psychological perception and satisfaction of the local affected people. Second, in terms of the remainder of the three candidates, the priority order derived from AHP was S3, S2, and S1, which was the reverse of the order calculated by the improved method. Judging from the psychological satisfaction of the local people when implementing the siting decision, the improved method’s conclusion is more acceptable and rational.

When the MCE method is applied to urban NIMBY facilities siting, the problem of qualitative criteria evaluation needs to be solved effectively. As a result, serious shortcomings exist in the current quantitative analysis methods. Researchers and practitioners can hardly make comprehensive and reasonable assessment on the siting factors with aforementioned method. The F function algorithm optimisation method proposed in this article can potentially improve the mixed MCE method and make a comprehensive and quantitative siting analysis. From the methodological view, the key to the F function optimisation is to change the nature of its signum function so that the magnitude of the evaluation value of the qualitative criterion can correlate with the calculation result. However, replacing the symbolic function with a linear function is a relatively simple way,and other function optimisation methods can be further explored. A successful resolution of the qualitative criteria evaluation can be made on the basis for both the decision-making process optimisation and the modular evaluation criteria system development. The optimisation and development together constitute the main elements of the mixed MCE method. We argue that three issues need to be reemphasised in terms of applying this mixed method to improve psychological satisfaction.

The first is the issue of cross-domain collaboration. NIMBY facilities siting is not a purely technical but a technical-social issue that involves various stakeholders. The collaboration of experts from different research domains and affiliations helps comprehensively consider the important factors affecting siting which are usually neglected, such as the residents’ psychological attitudes and assessment. This process can effectively reduce the probability of making mistakes in siting and the risk of social conflict. As Hung [42] pointed out, risk assessment from traditional expert perspective could not clearly answer the following questions: which factors were related to residents' risk attitudes and why risk perceptions and attitudes differed among residents. Furthermore, expert system's view of risk often ignored the differences in residents’ risk attitudes in different regions, which were formed by their divergent social backgrounds and psychological preferences.

The second issue is to define the evaluation scope of negative externality effects regarding NIMBY facilities. As an important basis for measuring criteria, this defining is particularly relevant to the evaluation of three criteria modulars, namely spatial planning, environmental protection, and socio-psychology. We argue that scholars should be aware of the environmental impacts that are not only caused by the NIMBY facilities themselves, but also by some intermediate links. For instance, waste treatment facilities, like landfills or incinerators, involve negative effects arising from the waste transport process. Thus, the possibility of including the waste transport routes should be considered when scoping the negative externality effects. In addition, scholars should also be aware that the public perception’s scope does not coincide with the negative externality effects’ scope. Public perception is an assessment of the intensity of public psychological satisfaction/dissatisfaction and acceptance/resistance and is also directly related to the public's risk perception. Actually, the public groups that are really involved and determined the course of the whole siting issue are actually the residents of several communities located outside the spatial scope of the environmental impact assessment. Their concerns about air pollution and the depreciation of their property value are the main force that drive them involved in the siting issue. For such facilities, expanding the spatial scope of public perception assessment needs to be considered on a case-by-case basis.

The third is the issue of myopic tendency of siting decision-making. Many NIMBY facility siting analyses are research which is conducted in actual projects or are related to public policymaking. The myopic decision preference of the decision-makers in these projects or public policymaking is caused by two reasons. First, when making decision, the makers often prefer emphasising on immediate interests and short-term goals. This decision preference contradicts the comprehensiveness and long-term tendency of NIMBY facility planning. Thus, it influences the formulation of the criteria and weights setting in the analysis model. Furthermore, residents’ psychological dissatisfaction often stems from the improvidence of the local government’s decision making, like selecting sites near the neighbourhoods which are vulnerable to political or economic issues. Second, myopic decision making is also caused by a unique Chinese political phenomenon, namely the local officials or cadres’ turnover in different regions, which is nearly accompanied with each change in leadership. Cadres’ frequent turnover implies the discontinuity in policy implementation as the decisions, public policies and plans made by the previous government are likely to be halted by the successive government. This phenomenon also determines the myopic decision making relate to public affairs, like NIMBY facility siting. Therefore, it easily declines the local residents’ psychological satisfaction.

## Conclusions

In view of the complexity of the factors and interests involved in urban NIMBY facilities siting, using a mixed MCE method to make decision is necessary to improve residents’ psychological satisfaction [43]. Although MCE is widely used in multi-site comparison analysis, the evaluation issue of qualitative criteria has yet been effectively solved, which greatly restricts the applicability of this method. A major issue is the over-emphasis of the physical factors and the neglection of social and psychological factors in the initial stage of evaluation. In responding to this issue, we argue to develop a criteria system through modularisation and introduce four modules, namely spatial planning, environmental protection, construction-operation, and socio-psychology. Among the four modules, socio-psychology module involves public opinion, psychological cognition, psychological assessment, public administration and management, and concentrates on public perception and policy impact. Accordingly, we regard public perception as the evaluation criterion of the socio-psychological module.

This paper explores a new MCE method, developed through interdisciplinary perspective between sociology and psychology study, to solve the insufficient consideration of urban spatial development, social public opinion, and psychological factors in the process of optimum siting of urban NIMBY facilities. Supported by this method, we pre-process the qualitative criteria by min–max standardisation and normalisation, turning all negative factors to positive, and transforming the F function into linear functions. The method can reasonably assign values to different combinations of qualitative criteria and expand the method’s potential application scope. Based on the effective solution to the qualitative criteria evaluation problem, we further optimise the siting decision process consisting of two stages of feasibility evaluation and MCE. Meanwhile, we propose a modular evaluation criteria system construction strategy from the perspective of cross-domain collaboration. In detail, we come to the following conclusions.

First, the F-function algorithm optimization proposed in this article greatly improves the qualitative and quantitative MCE, making comprehensive and quantitative multi-site optimization analysis possible. From a methodological perspective, the key to optimizing the F function is to change the properties of its sign function, so that the evaluation value of the qualitative criterion can be correlated with the calculation results. Thus, when MCE is applied to the optimal decision-making of multi-site selection for NIMBY facilities, the evaluation issue of qualitative criteria can effectively be solved. This greatly contributes to a comprehensive and reasonable evaluation of site selection considerations, leading to improvements in the current quantitative analysis methods and higher psychological satisfaction.

Second, the MCE for enhancing psychological satisfaction consists of two parts: qualitative criteria evaluation, and decision process optimization and modular evaluation criteria system construction. Specifically, we can obtain the following sub-conclusions: (1) The analysis process is divided into two stages: feasibility evaluation and MCE. The focus of the feasibility assessment stage is to develop an evaluation criteria system, distinguish between regulatory and guiding criteria, and determine candidate sites for subsequent evaluation through location feasibility screening. The work content of the MCE stage is to conduct a comprehensive evaluation of guiding criteria and analyze the selection of candidate sites. (2) The foundation of the MCE is the qualitative and quantitative method. By standardizing and normalizing the qualitative criteria, the evaluation method of the qualitative criteria is improved to effectively evaluate the superiority of the combination of qualitative criteria. (3) Based on the analysis of the planning and construction conditions of neighboring facilities, we can construct an evaluation criteria system in four modules: spatial planning, environmental protection, construction and operation, and social and political aspects. This can improve the level of collaborative decision-making among multiple disciplines and helps to evaluate the psychological satisfaction of residents. (4) A subjective and objective comprehensive weighting method should be adopted to improve the rationality of weight assignment.

Third, our empirical results indicate that the site selection of NIMBY facilities is an interactive process between society and nature involving multiple subjects, and the psychological satisfaction of residents towards it has great plasticity. This indicates that the psychological satisfaction of NIMBY facilities is generated by the dual logic of multi-module scene system arrangement and micro influencing factors. This further indicates that the governance strategies for psychological satisfaction of neighbor avoidance facilities not only require detailed exploration of precise evaluation criteria design from a micro level to shape public attitudes, but also require in-depth analysis of how to enhance the governance capacity and legitimacy of neighbor avoidance risks by strengthening the construction of evaluation modules from a macro level scenario planning (evaluation modules). Therefore, this also provides a strong reference for reshaping the decision-making mode of location selection for NIMBY projects. The design of our multi-module evaluation system indicates that the planning model formed by a highly closed, top-down decision-making model led by local government agencies may be questioned by residents around the site in terms of rationality and legality. Especially, the psychological satisfaction of the public cannot be incorporated into the decision-making of NIMBY projects, which will exacerbate the dilemma of public acceptance of NIMBY facilities. Thus, in the design of evaluation modules of NIMBY facilities, the multi-level decision-making for the planning and site selection of NIMBY projects should first clarify the stakeholders and their role positioning. For example, in our case, the stakeholders involved in the decision-making of NIMBY project generally include local government agencies, project planning and construction parties, technical experts, environmental organizations, and the public around the proposed site. Especially, as stakeholders in project decision-making, residents around the proposed site should participate rationally in decision-making. Moreover, the formation of a consensus based on MCE model for NIMBY projects requires dialogue and negotiation among multiple participating parties on the basis of equality and mutual respect. This also indicates the need for corresponding weight design. Overall, this consensus model of MCE can not only generate a site selection plan that is accepted by multiple parties, but also promote local governments to continuously engage in social learning and enhance the cohesion and cohesion among the policy network entities of waste to energy projects. Ultimately, such a MCE model will enhance the psychological satisfaction of residents around the proposed site and truly drive project planning of NIMBY facilities.

Our improved method is applied in the case study and shows its advantages in comparing with the AHP method in the local’s sanitary landfill siting. The mixed MCE rules out two candidates, which obviously contradicted with the urban special development priority and the psychological perception and satisfaction through feasibility evaluation, and alleviate the difficulty on rational siting. The priority order of the remainder candidates, as calculated by the improved MCE which is based on the modularisation criteria system, is also just the reverse of the result from the traditional method. Therefore, the case study implies that the improved method could be broadly applied to optimum siting decision of urban NIMBY facilities and effectively enhance the degree of public acceptance and their psychological satisfaction. Potential social conflicts in NIMBY facility siting will be reduced. In addition, the study provides a comprehensive and rational evaluation of siting schemes and optimises the location decision through two-stage location decision and building a modular criteria system. We acknowledge that there is no ‘one best way’ management or decision making [44, 45] in the NIMBY facility siting decision, as revealed by the studies of contingency theory in the realm of management study. The final decision on NIMBY facility siting hinges on various contingent factors, in particular the uncertain and instable context factors [44]. A potential of future study on NIMBY facility siting from the perspective of environmental psychology need to make allowance for the contingent factors and further improve the evaluation method and criteria system with contingency theory of management.

## Data Availability

The datasets generated and/or analysed during the current study are not publicly available due to the assurances in the informed consent agreement and ethic approval that the data will not be disclosed, but are available from the corresponding author on reasonable request.
